# Low‐Dose Melatonin, Climacteric Symptoms and Sleep in Female Shift Workers: A Randomized Controlled Trial

**DOI:** 10.1111/jpi.70140

**Published:** 2026-03-17

**Authors:** Susy P. Saraiva, Carolina V. R. D'Aurea, Cristina S. S. Luz, Fernanda G. Amaral, Jose Cipolla‐Neto, Elaine C. Marqueze, Claudia R. C. Moreno

**Affiliations:** ^1^ Department of Health and Society, School of Public Health University of São Paulo São Paulo Brazil; ^2^ Graduate Program in Collective Health Catholic University of Santos Santos Brazil; ^3^ Pineal Neurobiology Laboratory Federal University of São Paulo São Paulo Brazil; ^4^ Graduate Program in Public Health Federal University of Rio Grande do Norte Natal Brazil

**Keywords:** climacteric women, Hormonal fluctuations, melatonin administration, night shift workers

## Abstract

Sleep disturbances and mood changes are common during the climacteric, often linked to hormonal fluctuations caused by reduced ovarian function. Evidence suggests that long‐term melatonin administration may improve the quality of life in climacteric women. This study aimed to evaluate the effects of exogenous melatonin on climacteric symptoms, sleep quality, and reproductive hormones in women working fixed shifts. A randomized clinical trial was conducted with 46 nurses working fixed shifts at a hospital in São Paulo: morning (7:00–13:00, *n* = 16; intervention = 7, placebo = 9), afternoon (13:00–19:00, *n* = 15; intervention = 8, placebo = 7), and night (19:00–7:00, *n* = 15; intervention = 7, placebo = 8). Participants were randomly assigned to receive either 0.3 mg of melatonin or placebo. For night shift workers, melatonin was administered only on nights off, when they were sleeping at home; the same procedure was applied to morning and afternoon workers. Data collection included sociodemographic information, self‐reported sleep quality, and menopausal symptoms. Blood samples were collected at home to measure luteinizing hormone (LH), follicle‐stimulating hormone (FSH), estradiol, and progesterone before and after the intervention. A significant main effect of melatonin was observed, with a 15.8% reduction in climacteric symptoms compared with placebo (*p* = 0.01), independent of age or sleep duration, while no significant interaction with work shift was detected. Sleep quality improved by 35.33% on days off in the intervention group (*p* < 0.001), with morning shift workers showing a 32.46% improvement (*p* < 0.05). No significant changes were observed in reproductive hormone levels or total sleep duration. Exogenous melatonin effectively alleviates climacteric symptoms and improves sleep quality on days off, particularly among day‐shift workers, without affecting reproductive hormone concentrations or total sleep duration.

**Trial Registration:** RBR‐10whktxm (UTN: U1111‐1305‐6221). Registered on 13 August 2025, retrospectively registered.

## 1 Introduction

The climacteric involves pathological and physiological changes mainly due to declining ovarian function before and after the last menstruation [1]. According to the World Health Organization, menopause refers to the absence of menstruation for twelve consecutive months, usually between 45 and 55 years of age, resulting from ovarian follicular depletion that interrupts ovulation and menstrual regularity [2]. Ovarian aging is marked by reduced follicular reserve and elevated pituitary gonadotrophins, luteinizing hormone (LH) and follicle‐stimulating hormone (FSH) [3]. Despite the vast number of studies on the menopause, some aspects are still not fully understood. For example, gaps in knowledge on the menopausal transition and its impact on health remain [4]. While the biological process is the same for all individuals who menstruate, the experience of the menopause can differ [5].

In older women, night‐time levels of melatonin steadily decrease, whereas gonadotrophin levels constantly increase [6]. In addition, women undergo metabolic fluctuations, sleep disturbances and vasomotor symptoms during the climacteric [7]. Vasomotor symptoms, which include hot flashes and night sweats, are the main signs of the climacteric syndrome and affect women worldwide [8]. These symptoms, however, vary in frequency, duration and intensity [4]. Changes in sleep architecture during the climacteric and decrease in melatonin production with aging, increases the incidence of sleep disturbances [9, 10].

Melatonin, primarily produced by the pineal gland, follows a circadian rhythm regulated by light exposure and the suprachiasmatic nuclei [11, 12]. It is also synthesized in other organs and in the reproductive and immune systems [13, 14]. Light at night suppresses its production [12, 15]. Melatonin acts as a potent antioxidant, modulating oxidative stress and proinflammatory cytokines [13], and is linked to mood, sleep quality, and cardiovascular protection [16]. It also influences reproductive function, regulating GnRH, progesterone synthesis, and cytokine secretion [7, 17].

Scientific evidence suggests that melatonin benefits perimenopausal and menopausal women. In a randomized study, it improved depression, mood, and reduced FSH levels [6]. Over a decade later, a trial with women experiencing severe climacteric symptoms showed that administration of 3 mg of melatonin for 3 months led to significant improvements across psychological, somatic, vasomotor, and sexual domains [18]. Furthermore, a recent study confirmed effects on mood, sleep, quality of life, and bone protection [19]. In the same year, another trial with 60 postmenopausal women reported reductions in hot flushes, sleep disturbances, and headaches, supporting its use as a safe adjuvant therapy [20]. Lastly, a systematic review concluded that melatonin improves sleep and climacteric symptoms, with excellent safety [21].

Melatonin administration studies are particularly relevant for shift workers, as night shifts suppress melatonin production and lower circulating levels [22]. One study found 15% lower salivary melatonin in night workers on workdays, but not on days off [23]. Night‐time light exposure and melatonin suppression were also linked to irregular and shorter menstrual cycles in women working nights [24]. In this context, the negative impact of night work on women's health has become increasingly clear [25, 26]. Women working night shifts are at higher risk of developing chronic conditions, such as cardiovascular diseases [27, 28], type 2 diabetes [29] and of adverse impact on outcomes of pregnancy, such as stillbirths, pre‐term delivery and small‐for‐gestational age babies [30].

Building on evidence that long‐term melatonin administration alleviates climacteric symptoms, we hypothesize that these effects may be particularly significant among shift workers, due to their increased circadian misalignment. Therefore, the objective of the present study was to assess the effects of exogenous melatonin on climacteric symptoms, sleep and reproductive hormones in fixed‐shift workers and investigate differences in outcomes between day and night‐shift workers. Despite the positive results reported with higher doses, we chose a physiological dose of 0.3 mg to investigate whether even low‐dose melatonin could exert beneficial effects on the psychological, somatic, vasomotor, and sexual domains analyzed in this study.

## 2 Methods

The methodological details of this randomized, double‐blind, placebo‐controlled clinical trial have been previously described by Luz et al. [31], in a publication that focused exclusively on the effects of melatonin on dietary intake. The present article reports the trial's primary outcomes, including climacteric symptoms, sleep duration, and concentrations of reproductive hormones (LH, FSH, estradiol, and progesterone). Only the methods specific to these analyses are described below.

### 2.1 Study Design and Participants

A double‐blind randomized clinical controlled trial with placebo was conducted at a large hospital in São Paulo city, Brazil. Data collection took place between September 2022 and September 2023. The study population comprised 46 women in the climacteric who worked fixed‐shifts. Participants worked one of 3 shifts at the hospital: morning (7:00–13:00 h, *n* = 16), afternoon (13:00–19:00 h, *n* = 15) and night (19:00–7:00 h, *n* = 15). The shift rotor was 6 h on, 18 h off for the day‐time shifts, and 12 h on, 36 h off for the night shift. This study was registered at the Brazilian Registry of Clinical Trials (ReBEC) under the number RBR‐10whktxm (UTN: U1111‐1305‐6221), on 13 August 2025, and was retrospectively registered.

### 2.2 Sample Calculation

The sample size calculation was performed a priori using the software G*Power (version 3.1.9.7), based on an analysis of variance (ANOVA) for repeated measures with within–between interaction. The parameters adopted were an effect size (f) of 0.30, alpha error probability of 0.05, and statistical power (1–β) of 0.80. The design included six groups and two measurement points (pre‐ and post‐intervention), assuming a correlation of 0.5 among repeated measures and a nonsphericity correction (ε) of 1.0. Under these conditions, the total sample size required was 42 participants. Considering a potential attrition rate of 15%, the adjusted target sample size was 48 participants. The final sample comprised 46 women, consistent with the estimated parameters and resulting in an achieved power of approximately 81%. The effect size of 0.30 was adopted according to Cohen's conventional classification for medium effects in behavioral and biomedical research [32].

### 2.3 Intervention and Placebo Administration

Participants took a capsule containing 0.3 mg of melatonin (Intervention Group) or a placebo (Control Group) 1 h before their habitual bedtime over a 3‐month period. Night‐shift workers took the capsule on nights off, on alternating nights, while daytime workers (morning and afternoon shifts) took the capsule on alternating nights to match the dosing frequency of the night‐shift workers. In total, participants received melatonin or placebo on 45 nights throughout the 3‐month period. As the study was double‐blind, allocation to the intervention or placebo group was determined by a simple random draw conducted by a team member who was not involved in data collection.

### 2.4 Flow Diagram of Data Collection Process

**Figure 1:**
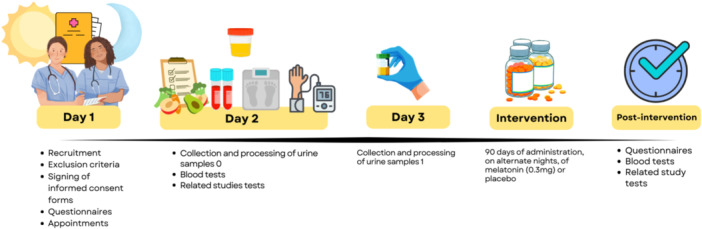
Flowchart of study procedures and data collection. Diagram illustrating the sequence of study procedures, including recruitment, biological sample collection, intervention period, and post‐intervention assessments.

The flow diagram depicting the stages of data collection, including recruitment, screening, biological (urine and blood) sample collection, application of questionnaires, and the 90‐day intervention with melatonin or placebo, followed by post‐intervention assessments, is presented in Figure 1.

### 2.5 Data Collection

Climacteric symptoms were assessed using the Kupperman Menopausal Index (KMI), an 11‐item tool rated on a severity scale of 1–3 in order of importance. Scores on the KMI range 0–19, 20–35 and > 35, indicating the degree of severity of symptoms and classified as mild, moderate and severe, respectively [33]. The Pittsburgh Sleep Quality Index (PSQI) was applied to rate overall quality of sleep based on a total score of 0 to 21. The Munich Chronotype Questionnaire (MCTQ) is a tool for assessing sleep patterns on work days and days off, to determine an individual's chronotype and sleep time [34]. In the present study, the data for sleep duration on days off were employed. The above‐mentioned scales were applied to all participants both before and after the intervention. Fasting blood samples were collected from all participants at their homes by venous puncture, pre and post‐intervention, at a pre‐arranged time between 06:00 and 08:00. A third‐party specialized company hired for the study carried out the collection procedures to determine levels of reproductive hormones (LH, FSH, E2 and progesterone).

This study was part of a larger research project whose data gathered (Figure 1) were used in other studies exploring different outcomes among women during the climacteric, such as body weight [31].

### 2.6 Statistical Analysis

The software package Stata 16 (StataCorp, 2019) was used for all statistical treatment, together with the software GraphPad Prism (version 8, GraphPad Software) and Jamovi (version 2.3.28) to produce the graphs and tables used. Between‐group comparisons of demographic and clinical data at study baseline were performed using the *t*‐test for independent samples or Fisher's exact test. For the analysis of post‐intervention data, the *t*‐test for independent samples and the paired *t*‐test for between‐group and within‐group comparison were employed, respectively. Analyses of variance (ANOVA) were performed to identify possible differences between the shifts.

**Figure 2:**
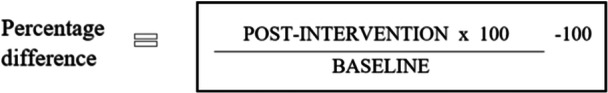
Formula for calculating percentage difference. Mathematical formula used to determine the percentage change between post‐intervention and baseline values. The result represents the relative increase or decrease after the intervention.

In cases where the assumption of normality was not met, the generalized linear model (GLM) with 3 factors (shift, group and menopausal status) was applied, and the percentage difference between pre and post‐intervention (Figure 2) was defined as the outcome variable and calculated for all parameters. The percentage difference was calculated to quantify the increase or decrease in percentage terms, aiding comparison of changes between different time points of the study. A positive value shows an increase relative to baseline, whereas a negative value reflects a decrease. A percentage of zero indicates no change in the post‐intervention value compared to baseline. Sleep duration on days off at the baseline was used as adjustment variable, with a cut‐off point of 6 h, as determined in the study by Gusmão et al. [35].

The level of significance was set at *p* < 0.05 for all statistical tests. Post‐hoc analyses with Bonferroni adjustment was carried out to identify statistically significant between‐group differences. Age was included as a covariate in all generalized linear models due to its known influence on melatonin secretion, sleep parameters, and climacteric symptoms. Descriptive tables report raw means and standard deviations for sample characterization only and should not be interpreted as measures of intervention effectiveness. All inferential analyses regarding treatment effects are based on adjusted generalized linear models comparing intervention and placebo groups.

### 2.7 Ethics Aspects

All study participants were provided with detailed information and granted written informed consent to participating before inclusion in the study. This study was approved by the Research Ethics Committee of the hospital where data collection took place, under permit no. 5.658.804, on 22/09/2022, and by the Research Ethics Committee of the School of Public Health of the University of São Paulo (FSP/USP), under permit no. 5.468.644, on 14/06/2022.

## 3 Results

### 3.1 Demographic Characteristics

Participants had a mean age of 47.2 ± 5.4 years (range = 39–60 years) at the time of data collection. The majority of participants was nursing technicians (73.9%), had secondary level education, concluded technical training (57.4%), and had an income of between R$ 3601.00 and 5400.00 (65.9%). Most of the individuals in the sample were married (54.3%) and had children (84.7%) at data collection. Less than half (34.7%) of the participants had gone through the menopause. In terms of race by shift type, 50% of the morning shift workers were white and 50% black (black and brown), whereas 53.3% on the afternoon shift were white and 46.7% black. The night shift was the only shift that the participants were predominantly (53.3%) black. On the group analysis by shift, significant age difference was evident only for the night shift (*p* < 0.01) (Table 1). No significant baseline differences were observed between intervention and placebo groups regarding smoking status or napping habit, either within shifts or across work shifts (Table 1).

**Table 1:** Demographic characteristics of participants (*n* = 46), according to work shift and groups.

Demographic characteristics	Morning shift (*n* = 16) Mean ± SD		*p* value^a^	Afternoon shift (*n* = 15) Mean ± SD		*p* value^a^	Night shift (*n* = 15) Mean ± SD		*p* value^a^	*p* value (shifts)^b^
	Intervention group (*n* = 7)	Placebo group (*n* = 9)		Intervention group (*n* = 8)	Placebo group (*n* = 7)		Intervention group (*n* = 7)	Placebo group (*n* = 8)		
Age (year)	49.93 ± 6.5	45.06 ± 4.34	0.08	47.06 ± 5.73	48.51 ± 5.87	0.63	43.03 ± 3.51	50.02 ± 4.93	**0.008** **	0.78
Race (%)										
White	28.57	66.67	0.31	50.0	57.14	1.00	28.57	50.0	0.60	0.81
Non‐white (black, brown and yellow)	71.43	33.33		50.0	42.86		71.43	50.0		
Job position (%)										
Nurse	28.57	22.22	1.00	0	42.86	0.07	28.57	37.50	1.00	0.77
Nursing Technician	71.43	77.78		100	57.14		71.43	62.50		
Menopausal Status (%)										
Yes	42.86	33.33	1.00	12.5	42.86	0.28	42.86	37.5	1.00	0.79
No	57.14	66.67		87.5	57.14		57.14	62.5		
Smoking (%)										
Non‐smoker	100	88.89	1.00	100	85.71	0.46	85.71	75	1.00	0.50
Ex‐smoker	0	11.11		0	14.29		14.29	25		
Napping habit (%)										
Yes	85.71	77.78	1.00	50.0	57.14	1.00	85.71	87.5	1.00	0.14
No	14.29	22.22		50.0	42.86		14.29	12.5		

*Note:* Data are presented as mean ± (SD) for continuous variables and as percentages for categorical variables. Differences were considered statistically significant at *p* < 0.05; *p* < 0.01 indicates high statistical significance. *p < 0.05.

^a^ *t*‐test for independent samples and Fisher's exact test.

^b^ Two‐Way ANOVA (group and shift; *p*‐value of shift) and Fisher's exact tests.

** *p* < 0.01.

### 3.2 Baseline Sleep Characteristics

Differences in sleep patterns among workers according to shift (morning, afternoon and night), work days and days off, and by group (intervention and placebo), are presented in Table 2. On days off, afternoon shift workers had a tendency to sleep later compared to morning shift workers, in both the intervention and placebo groups. This disparity might be due to differences in routine work times. However, no major difference for these two shift types versus the night shift were evident on days off. On work days, night shift workers had a tendency to sleep much later compared to those on the morning shift, in both placebo and intervention groups. A similar difference was seen between night and afternoon shifts, showing that night work had a substantial impact on sleep onset time (Table 2). There was a significant difference in workday wake‐up time across shifts. Night‐shift workers woke up much later, whereas those on the morning shift woke up earlier, in both the intervention and placebo groups. Afternoon shift workers had an intermediate wake‐up time in both groups, showing a significant difference compared to both morning and night shifts (Table 2).

**Table 2:** Sleep onset, wake‐up time, and sleep duration on workdays and days off at baseline, according to groups and work shifts (*n* = 46).

	Morning shift (*n* = 16) Mean ± SD		*p* value^a^	Afternoon shift (*n* = 15) Mean ± SD		*p* value^a^	Night shift (*n* = 15) Mean ± SD		*p* value^a^	*p* value (shifts)^b^
	Intervention group (*n* = 7)	Placebo group (*n* = 9)		Intervention group (*n* = 8)	Placebo group (*n* = 7)		Intervention group (*n* = 7)	Placebo group (*n* = 8)		
Day‐off sleep onset (time)	22:23 ± 36 min	22:51 ± 1 h 06 min	0.33	00:12 ± 1 h 42 min	23:35 ± 1 h 36 min	0.48	23:50 ± 1 h 10 min	23:03 ± 1 h 05 min	0.19	**0.027** *
Day‐off wake‐up (time)	6:43 ± 1h36min	7:09 ± 1 h 43 min	0.61	8:37 ± 2 h 16 min	7:47 ± 51 min	0.37	7:39 ± 1 h 58 min	7:22 ± 1 h 51 min	0.79	0.15
Day‐off sleep duration (hours)	8 h 19 min ± 1 h 30 min	8 h 18 min ± 2 h 22 min	0.98	8 h 10 min ± 2 h 22 min	8 h 18 min ± 1 h 43 min	0.91	8 h 57 min ± 1 h 04 min	8 h 20 min ± 2 h 10 min	0.50	0.83
Workday sleep onset (time)	22:15 ± 42 min	22:41 ± 1 h	0.34	23:27 ± 1 h 11 min	23:31 ± 55 min	0.91	9:52 ± 1 h 16 min	10:36 ± 1 h 48 min	0.4	< **0.001** **
Workday wake‐up (time)	5:03 ± 36 min	4:34 ± 22 min	0.06	7:34 ± 1 h 46 min	7:04 ± 44 min	0.5	12:13 ± 1 h 16 min	14:41 ± 2 h 44 min	0.06	**< 0.001** **
Workday sleep duration (hours)	6 h 31 min ± 1 h 21 min	6h 07 min ± 54 min	0.49	8h 14 min ± 1 h 58 min	7 h 33 min ± 1 h 17 min	0.45	3 h 18 min ± 3 h 04 min	4 h 04 min ± 1 h 48 min	0.55	< **0.001** **

*Note:* Values are presented as mean ± (SD).

^a^ Independent *t*‐test used.

^b^ Two‐way ANOVA with shift and group as factors.

* *p* < 0.05

** *p* < 0.01.

Lastly, there were significant differences between the night shift and other shifts in terms of sleep duration on work days in both groups. Mean sleep duration was significantly shorter for the nightshift compared to both morning and afternoon shifts. This finding indicates individuals who worked the night shift slept less on work days than those engaged in morning and afternoon shifts, reflecting the difficulty of this group in maintaining a stable sleep pattern (Table 2).

### 3.3 Climacteric Symptoms

There was a moderate prevalence of vasomotor symptoms, characterized by hot flashes and sweats, in the sample at baseline. Of the participants assessed, 43.48% reported no symptoms, 36.96% mild symptoms, and 19.57% moderate severity. There were no severe cases in the sample (Table 3). Paresthesia, vertigo, palpitation and formication were the least reported symptoms. By contrast, the most severe symptoms included insomnia, nervousness, melancholia and weakness/fatigue, with a considerable proportion of participants reporting mild‐to‐moderate levels of these symptoms (Table 3). There were no group or shift differences in climacteric symptoms prior to the intervention (Table 4).

**Table 3:** Distribution of 46 participants according to symptoms, prevalence and severity, as classified by KMI at baseline.

Symptoms	Absent		Mild		Moderate		Severe	
	*n*	%	*n*	%	*n*	%	*n*	%
Vasomotor	20	43.48	17	36.96	9	19.57	0	0
Paresthesia	28	60.87	13	28.26	5	10.87	0	0
Insomnia	17	36.96	12	26.09	15	32.61	2	4.35
Nervousness	9	19.57	16	34.78	19	41.30	2	4.35
Melancholia	12	26.09	20	43.48	14	30.43	0	0
Vertigo	29	63.04	13	28.26	3	6.52	1	2.17
Weakness/Fatigue	10	21.74	20	43.48	11	23.91	5	10.87
Arthralgia and/or Myalgia	12	26.09	15	32.61	11	23.91	8	17.39
Headache	12	26.09	17	36.96	12	26.09	5	10.87
Palpitations	28	60.87	16	34.78	2	4.35	0	0
Formication	29	63.04	13	28.26	3	6.52	1	2.17

*Note:* Data are presented as absolute numbers (*n*) and percentages (%) of participants classified by symptom severity according to the Kupperman Menopausal Index (KMI) at baseline. Severity levels are categorized as absent, mild, moderate, or severe.

**Table 4:** Climacteric Symptoms KMI, according to group and shift, at baseline and post‐intervention (*n* = 46).

	Morning shift (*n* = 16) Mean ± SD		*p* value	Afternoon shift (*n* = 15) Mean ± SD		*p* value	Night shift (*n* = 15) Mean ± SD		*p* value	*p value* a
	Intervention group (*n* = 7)	Placebo group (*n* = 9)		Intervention group (*n* = 8)	Placebo group (*n* = 7)		Intervention group (*n* = 7)	Placebo group (*n* = 8)		
Baseline Climacteric Symptoms	18.7 ± 6.7	13 ± 10.7	n.s	15.9 ± 9	13.7 ± 9.5	n.s	13.6 ± 6.3	15.2 ± 7.3	n.s	0.88
Post‐intervention Climacteric Symptoms	14.3 ± 8	14.2 ± 10	n.s	17.6 ± 10.6	13.4 ± 8.7	n.s	13.8 ± 5.5	20 ± 10.3	n.s	0.73

*p < 0.05, **p < 0.01.

^a^ Two‐way ANOVA. Factors: shift and group. *p*‐value for shift factor alone.

Results of the analysis of percentage difference between pre‐ and post‐intervention, including work shift, group, and menopausal status, and adjusted for age, sleep duration on days off at baseline, anxiety, stress, and depression, showed a mean reduction in symptoms of 15.8% in the melatonin group, representing a significant difference compared with the placebo group, which showed a mean increase of 33.5% (*p* = 0.01) (Table S1). This reflects an adjusted overall intervention effect, independent of work shift and baseline differences.

**Graph 1:**
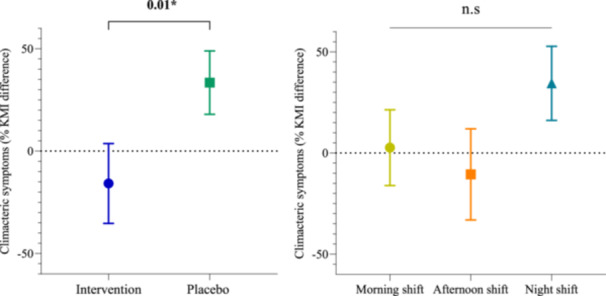
Percentage difference in Kupperman Menopausal Index between baseline and post‐intervention, by group and work shift (*n* = 46). The left panel shows the main effect of group (intervention vs placebo), independent of work shift, with the intervention group represented in blue and the placebo group in green. The right panel shows the main effect of work shift, independent of group, with morning shift workers indicated in yellow, afternoon shift in orange, and night shift in blue. Values represent adjusted mean percentage change in the Kupperman Menopausal Index (KMI) from baseline to post‐intervention, derived from generalized linear models. Error bars represent the standard error (SE) of the mean. A *p*‐value < 0.05 indicates a statistically significant main effect based on the adjusted model; “n.s.” indicates no statistically significant difference.

No significant main effects were observed across work shifts (*p* = 0.13) or menopausal status (*p* = 0.78) (Graph 1, 3; Table S1). However, a marginally significant interaction between group and menopausal status was identified (*p* = 0.05), indicating that postmenopausal women in the placebo group showed a marked increase in symptoms (58.04%), whereas those in the intervention group showed a reduction of 34.51%. These findings suggest a potential moderating role of menopausal status in the response to melatonin. Graph 1, 3 presents the main effect of group (intervention vs placebo) on the left panel, independent of work shift, and the main effect of work shift on the right panel, independent of group.

### 3.4 Sleep Duration – Days Off

**Figure 3:**
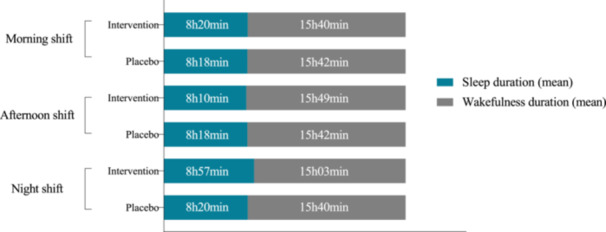
Baseline mean sleep and wakefulness durations on days off by group and work shift (*n* = 46). Bar graph showing the mean sleep duration (in blue) and wakefulness duration (in gray) on days off for participants in the intervention and placebo groups, stratified by work shift (morning, afternoon, night) at baseline. Values are presented in hours and minutes.

**Figure 4:**
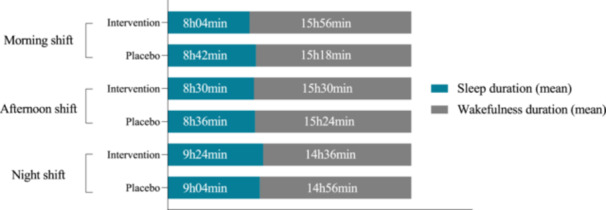
Post‐intervention mean sleep and wakefulness durations on days off by group and work shift (*n* = 46). Bar graph showing the mean sleep duration (in blue) and wakefulness duration (in gray) on days off for participants in the intervention and placebo groups, stratified by work shift (morning, afternoon, night) after the intervention. Values are presented in hours and minutes.

Although there was an increase in sleep duration on days‐off across practically all the groups and shifts (except intervention group morning shift) (Figures 1, 3 and 4), the percentage difference was not significant (Table 5).

**Table 5:** Generalized linear model (GLM) of the percentage difference in sleep duration on days off, adjusted for age (*n* = 46).

	Mean (%)	SE	*p‐value*
Group			
Intervention	12.2	6.83	0.955
Placebo	11.7	5.67	
Shift			
Morning	8.93	6.94	0.495
Afternoon	20.11	9.14	
Night	6.89	7.04	
Menopausal Status			
Yes	21.14	7.88	0.063
No	2.82	5.16	

*Note:* Mean percentage differences in sleep duration on days off are presented with standard errors (SE), based on a generalized linear model (GLM). The model includes the factors: work shift, experimental group (intervention or placebo), and menopausal status, adjusted for age. *p < 0.05, **p < 0.01.

### 3.5 Sleep Quality

There was no group or shift difference in sleep quality prior to the intervention (Table S2 of Supporting Information). Differences between work shifts were evident after the intervention (*p* < 0.001) for sleep quality on workdays (Table 6). Bonferroni analysis showed differences between the morning and night shifts (*p* = 0.002), while the difference between afternoon and night shifts was borderline (*p* = 0.06), specifically for sleep quality on workdays.

**Table 6:** Post‐intervention sleep quality on days off and workdays, by group and work shift (*n* = 46).

	Morning shift (*n* = 16) Mean ± SD		*p* value	Afternoon shift (*n* = 15) Mean ± SD		*p* value	Night shift (*n* = 15) Mean ± SD		*p* value	*p value* ^a^
	Intervention group (*n* = 7)	Placebo group (*n* = 9)		Intervention group (*n* = 8)	Placebo group (*n* = 7)		Intervention group (*n* = 7)	Placebo group (*n* = 8)		
Day‐off sleep quality	4.43 ± 1.4	5.44 ± 2.8	n.s	6 ± 2.4	6 ± 4.5	n.s	5.6 ± 3.7	5.5 ± 2.8	n.s	0.63
Workday sleep quality	4.85 ± 2	5.5 ± 2.8	n.s	6.4 ± 2.8	6.7 ± 4.2	n.s	8.8 ± 2.6	9.2 ± 1.4	n.s	**< 0.00** **

*Note:* Values are presented as mean ± (SD) for sleep quality scores on workdays and days off, in the intervention and placebo groups, stratified by work shift (morning, afternoon, night). Comparisons were performed using a Generalized Linear Model with group and shift as factors. *p*‐value^a^ refers to the main effect of the shift factor alone. *p < 0.05

** *p* < 0.01, n.s. = not significant.

**Graph 2:**
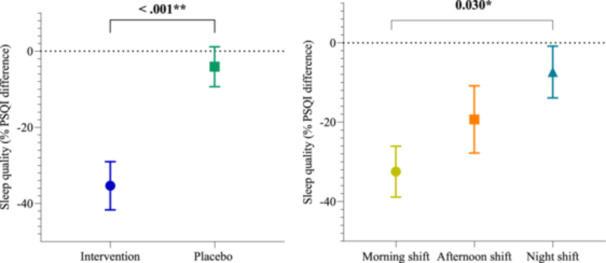
Percentage difference in Pittsburgh Sleep Quality Index (on days off) between baseline and post‐intervention by group and work shift, adjusted for age (*n* = 46). The left panel displays the main effect of group (intervention vs placebo), independent of work shift, with the intervention group represented in blue and the placebo group in green. The right panel displays the main effect of work shift, independent of group, with morning shift workers indicated in yellow, afternoon shift in orange, and night shift in blue. Values represent adjusted mean percentage change in Pittsburgh Sleep Quality Index (PSQI) scores on days off from baseline to post‐intervention, derived from generalized linear models. Negative values indicate improvement in sleep quality, whereas positive values indicate worsening. Error bars represent the standard error (SE) of the mean. A *p*‐value < 0.05 indicates a statistically significant main effect based on the adjusted model; “n.s.” indicates no statistically significant difference.

Results of the analysis of percentage difference between pre‐ and post‐intervention for the above‐mentioned factors, plus menopausal status, after adjusting for age, showed an isolated effect of group (*p* < 0.001) and shift (*p* = 0.023), but not menopausal status (*p* = 0.86), on sleep quality on days off (Table S3 of Supporting Information). Bonferroni analysis revealed differences between the morning and night shifts (*p* = 0.030) (Graph 2). Interactions were also observed between groups and shifts; groups and menopausal status; shifts and menopausal status; and groups, shifts and menopausal status (Table 7). Graph 2 displays the main effect of group (intervention vs placebo) on the left panel, independent of work shift, and the main effect of work shift on the right panel, independent of group.

**Table 7:** Generalized linear model (GLM) of Sleep Quality (PSQI) on days off, adjusted for age (*n* = 46).

	Difference	SE	*p*‐value	*p‐value post‐hoc* ^*a*^
SHIFT				
Morning x Afternoon	−13.2	10.53	**0.023** *	0.662
Morning x Night	−25.1	9.18		**0.030** *
Afternoon x Night	−12.0	10.79		0.828
G x S				
Intervention Morning x Placebo Afternoon	−47.64	13.4	**0.022** *	**0.017** *
Intervention Afternoon x Placebo Afternoon	−63.05	16.3		**0.007** **
Intervention Afternoon x Placebo Night	−55.85	16.0		**0.021** *
G x MS				
Intervention Menopausal x Placebo Menopausal	−63.48	13.93	**< 0.001** **	**< 0.001** **
Placebo Menopausal x Intervention Non‐menopausal	32.762	11.15		**0.036** *
Placebo Menopausal x Placebo Non‐menopausal	33.756	10.48		**0.017** *
S x MS				
Morning Menopausal x Night Menopausal	−48.91	14.3	**< 0.001** **	**0.025** *
Morning Non‐menopausal x Afternoon Non‐menopausal	−38.26	11.1		**0.023** *
Afternoon Menopausal x Night Menopausal	−60.86	18.1		**0.030** *
Afternoon Non‐menopausal x Night Non‐menopausal	36.96	11.3		**0.039** *
Night Menopausal x Morning Non‐menopausal	51.29	13.0		**0.006** **
Night Menopausal x Night Non‐menopausal	50.00	13.3		**0.010** *
G x S x MS			**0.045** *	

*Note:* Comparisons of sleep quality on days off using a generalized linear model (GLM), adjusted for age. The analysis includes main effects and interactions among work shift (S), group (G), and menopausal status (MS). Significant pairwise comparisons were further explored using Bonferroni post hoc tests^a^. Results are expressed as mean differences, standard errors (SE), and *p*‐values.

* *p* < 0.05

** *p* < 0.01.

### 3.6 Hormonal Level

The analysis of percentage difference revealed no isolated group or shift effects on LH, FSH, E2 and progesterone levels. Similarly, no effects on interactions were evident. Results are presented in Table S4 of Supporting material.

## 4 Discussion

The current results revealed that a 0.3 mg dose of melatonin, similar to physiological serum concentrations, administered for 3 months on alternate nights, was effective in alleviating climacteric symptoms in women working fixed shifts. The 15.8% post‐intervention improvement remained significant after adjustment for sleep duration on days off, anxiety, stress, and depressive symptoms, indicating a robust intervention effect independent of these baseline factors.

At baseline, there was a moderate prevalence of vasomotor symptoms, with most participants reporting none or only mild manifestations; fewer than 20% had moderate symptoms, and no severe cases were observed. Thus, although relatively frequent, vasomotor symptoms tended to be mild. Paresthesia, vertigo, and palpitations were less common, while insomnia, nervousness, melancholia, and fatigue were more severe. This suggests that emotional and fatigue‐related symptoms, such as nervousness and weakness, were more prevalent and intense among nursing professionals, possibly due to the profession's emotional and physical demands. It is worth noting that the relatively mild symptom burden at baseline may have limited the magnitude of improvement observed following the intervention. Nevertheless, the difference between the intervention and placebo groups remained statistically significant, reinforcing the efficacy of melatonin even in a population with initially low symptom severity. Although differences between work shifts were not statistically significant, a trend emerged: morning and night shift workers had climacteric symptom increases of 2.63% and 34.4%, respectively, while afternoon workers showed a 10.5% reduction. This suggests greater circadian disruption in night workers may impair melatonin's effectiveness, while early waking may limit its effects in morning workers. Despite these descriptive trends across shifts, the adjusted analyses demonstrated a significant overall benefit of melatonin compared with placebo, indicating that the intervention effect was independent of shift‐related heterogeneity.

No significant differences were found between menopausal and non‐menopausal women (mean symptom increases of 11.7% and 5.93%, respectively), indicating that melatonin's efficacy is not restricted to menopausal status. However, a significant interaction showed that women who had already gone through menopause in the intervention group had a 34.51% reduction in climacteric symptoms, while those in the placebo group had a 58.04% increase (*p* = 0.05). Thus, although melatonin is beneficial regardless of menopausal status, its effects may be stronger after menopause due to specific physiological changes.

In menopausal women with depression, increased nocturnal melatonin secretion and a delayed timing of melatonin offset have been reported compared with healthy controls, suggesting circadian misalignment rather than melatonin deficiency. Higher melatonin levels and longer melatonin duration were associated with greater depressive symptom severity, eveningness, and later sleep end times [36]. Consistently, findings in postmenopausal women indicate that depression is associated with alterations in circadian parameters of melatonin. In particular, current major depression was significantly associated with a later offset of 6‐sulfatoxymelatonin excretion, accompanied by a tendency toward longer nocturnal secretion duration and a later acrophase compared with healthy controls. In addition, a positive family history of depression was significantly related to longer 6‐sulfatoxymelatonin excretion duration, suggesting a possible familial vulnerability in the endogenous melatonin signal and an abnormality in the duration of this signal in women with current major depression [37].

Previous studies have shown that, while the ideal sleep time for cardiovascular health and longevity appears to range from 6 to 8 h [38, 39], the duration differs significantly when sleep is measured subjectively versus objectively (e.g. by using actigraphy). Gusmão et al. using objective measurements, found that 5–6 h of sleep time was associated with better vascular characteristics in night‐shift workers [35]. In the present study, the cut‐off point of 6 h of sleep may have helped isolate a subgroup with insufficient sleep time. This distinction is important because both sleep quality and quantity play a key role in regulating the hormones and physiological processes that influence climacteric symptoms.

This finding corroborates previous studies showing that melatonin alleviates climacteric symptoms. Chen et al. reported that administering 3 mg of melatonin for 3 months to peri‐ and postmenopausal women with sleep disorders improved sleep quality and increased circulating melatonin, along with significant changes in FSH, LH, and estradiol, without affecting progesterone, testosterone, or prolactin. However, the study did not evaluate daytime melatonin levels (“escape”) or nighttime concentrations [40]. A systematic review concluded that doses of 3 mg or more improve one or more climacteric symptom domains, regardless of baseline severity. Most high‐quality studies in the review showed improvements after ≥ 3 mg of melatonin for at least 3 months [21]. Importantly, the studies in question used doses 10 times higher than those employed in the present study.

Other studies have also shown that melatonin administration has a positive effect on climacteric symptoms, sleep and hormone levels during the perimenopause, avoiding the drawbacks of hormone replacement therapy to some degree [20, 41, 42]. In the case of sleep, melatonin confers beneficial effects through soporific action and by helping to synchronize circadian rhythms [43]. The hormone can also favor sleep through vasodilatory and peripheral thermoregulatory actions. The hormone's effects are modulated by sex steroids, which may be low in post‐menopausal women [44], The effects of melatonin may be further reduced by early wake‐up times, as occurs in morning workers, and by exposure to light at night, as occurs in night‐shift workers, facilitating insomnia and other disorders. Moreover, changes in melatonin levels induce major changes in the biological functioning of almost all organs [45], with potential implications for sleep quality [43].

It is important to highlight that melatonin plays a central role in nocturnal thermoregulation in humans, exhibiting an inverse relationship with core body temperature and promoting distal cutaneous vasodilation, heat loss, and a reduction in core temperature, processes that are essential for sleep initiation and maintenance [11]. During the climacteric period, the decline in estrogen destabilizes the thermoregulatory system, narrows the thermoneutral zone, and increases the occurrence of hot flashes, characterized by abrupt cutaneous vasodilation and sweating [46]. Under conditions of circadian disruption, such as shift work, the melatonin rhythm may be suppressed or misaligned, affecting the hypothalamic–pituitary–ovarian axis [47] and exacerbating this thermoregulatory instability. Reproductive hormones modulate melatonin secretion and participate in the regulation of body temperature. Moreover, hormonal variations throughout the reproductive cycle and ovarian aging influence the pattern of melatonin secretion [48]. Thus, the interaction among melatonin, body temperature, and sex hormones represents a plausible physiological pathway underlying the co‐occurrence of sleep disturbances and vasomotor symptoms in climacteric women.

The present study findings showed that melatonin, administered at doses closely matching physiological sera concentration, improved subjective sleep quality in women working all shifts on days off, irrespective of age. The effect was especially marked in the group engaged in the morning shift, which showed a 32.46% improvement in symptoms. Based on these results, it can be inferred that shift type had a major influence on sleep outcomes in this group of workers, with a greater impact seen in those doing the morning shift than the night shift.

Pines (2016), in a study exploring health in women over 40 and its association with biological rhythms, posed the following questions: Is the menopause a manifestation of the aging process of circadian rhythms? and; Is the menopause affected by aging of the circadian rhythm? [49, 50]. In fact, the ovarian gonadotrophin release mechanism is indirectly regulated by neuronal stimuli, which promote the release of GnRH (Gonadotrophin‐Releasing Hormone) by the hypothalamus. This hormone, in turn, stimulates the pituitary gland to release LH and FSH, hormones responsible for regulating release of progesterone and estradiol by the ovaries. Interruption of this hormonal regulation with advancing age can lead to irregular menstrual cycles or even amenorrhea. The author also stated that sleep disturbances are a hallmark symptom of the menopause and holds that any deviation from the normal sleep‐wake circadian pattern is highly relevant [50].

The circadian rhythm of melatonin production by the pineal gland is an extensively investigated underlying mechanism that is tightly synchronized with habitual sleep cycle [51, 52]. There is a steady decline in melatonin production with aging across genders, but this is exacerbated among women due to the menopause, affecting sleep regulation [53]. Other factors, such as hot flashes, also influence sleep quality. In a study of symptomatic and asymptomatic women, a circadian pattern of hot flashes was identified, peaking at around 18:25p [54].

Aging is typically associated with both changes in the circadian system and reduced melatonin production, two strongly associated phenomena [43]. Reproductive and chronological aging are also closely intertwined, exerting interdependent (and often bidirectional) effects. These conditions are hard to assess in isolation because they invariably overlap. Epidemiological studies have revealed that the risk of dementia is higher in women, given the modulating role of sex steroids on cognition [55, 56]. Thus, loss of ovarian hormones during the menopause may act on cognitive reserve, both directly and indirectly, via increased sleep disturbances, speeding up cognitive decline [57].

Several studies have reported the influence of sex hormones on sleep [55, 58, 59, 60]. Progesterone has anxiolytic and sedative effects, promoting non‐REM sleep [43], while low estrogen is linked to more severe night‐time awakenings [61] and affects nocturnal body temperature regulation [62]. However, no consistent correlation has been found between polysomnographic data, menopausal stages, and hormone levels, likely due to varied methods and lack of standardization during the phases of the climacteric [43]. This complexity hinders isolated analyses of hormonal effects. For instance, in perimenopausal insomniacs, FSH was not associated with wakefulness after sleep onset [58], despite frequent insomnia and awakenings. In the present study, no group differences in LH, FSH, estradiol, or progesterone were observed before and after intervention, though cycle phase, uncontrolled here, can affect reference values.

Systematic reviews and meta‐analyses suggest that, despite the low number of randomized clinical trials involving menopausal women, melatonin appears to be a valuable treatment option for individuals in the menopause, representing a safe alternative to other drugs [52] and to hormone replacement therapy, given its good tolerability and absence of safety issues in concomitant therapy with anti‐hypertensive, anti‐diabetic and anti‐inflammatory agents [43]. There are few reports concerning the tolerance, dependency, or feelings of “hangover” associated with the use of exogenous melatonin, supporting its use as an effective alternative when indicated [63]. In the present study, melatonin was well‐tolerated and there were no reports of morning hangover or other collateral effects following use of the hormone.

Climacteric women naturally experience changes in sleep architecture and reduced melatonin production. These effects, intensified by shift work, can worsen climacteric symptoms. In this study, intermittent use of 0.3 mg melatonin over 3 months significantly improved self‐reported symptoms and sleep quality on days off among a population of workers in the climacteric. The results suggest melatonin may be a therapeutic option for managing symptoms and enhancing sleep in shift‐working women. Lastly, melatonin, although effective for regulating the sleep‐wake cycle, can be less effective in individuals whose sleeping hours are significantly misaligned with a normal daytime pattern. Longer interventions might be more effective in this group. Moreover, the lack of difference in sleep duration observed post‐intervention, relative to baseline, may also indicate that melatonin impacted the quality of sleep more than the amount.

Although the present study was not designed as an implementation trial, its findings are highly relevant from a translational and implementation science perspective. The use of a physiological dose of melatonin, administered on days off and aligned with participants’ work schedules and sleep‐wake patterns, addresses key barriers commonly observed in the translation of sleep and circadian interventions into routine practice. It is known that feasibility and adherence are critical determinants of effectiveness, often limiting the impact of interventions that demonstrate efficacy under controlled conditions.

Although the study population included different shift types, no significant interaction between intervention and work shift was observed, supporting an overall beneficial effect of melatonin across shifts. Nevertheless, descriptive trends suggest that circadian disruption may modulate individual responses, which warrants further investigation. Psychological and sleep‐related factors remain important contributors to the perception and severity of climacteric symptoms and should be further explored in future studies. This study has some limitations. It may be the case that an intervention longer than 3 months could potentiate the alleviation of climacteric symptoms seen in this study. Blood collection in participants who still menstruated was not standardized with respect to the phases of the menstrual cycle, a factor which might have influenced the hormone results. In addition, although melatonin administration was scheduled relative to each participant's habitual bedtime, chronotype was not formally assessed, therefore, it was not possible to explore whether chronotype moderated the response to melatonin. Also, these results cannot be extrapolated to other individuals in use of hormone therapy for gender transition. It should be noted that this is the first study assessing the effects of melatonin administration, at doses matching physiological sera concentrations, associated with sleep duration, on climacteric symptoms of night‐shift workers. Despite the limitations outlined, it is hoped these initial results can pave the way for future investigations.

## 5 Conclusions

The results of this study suggest that the administration of 0.3 mg of melatonin for 3 months significantly alleviated climacteric symptoms and improved sleep quality on days off in shift workers, irrespective of age. However, no statistically significant changes in reproductive hormone levels or sleep duration were evident after the intervention relative to baseline.

These findings also suggest melatonin administration can be an effective alternative therapy for alleviating symptoms of the climacteric in women working fixed shifts. Importantly, these results have implications beyond clinical management, highlighting the need for occupational health policies that consider the specific challenges faced by shift‐working women during the climacteric. By addressing sleep quality and climacteric symptoms in the workplace, interventions such as melatonin supplementation could contribute to improved well‐being, productivity, and overall quality of life among this population.

Nevertheless, future investigations involving more diverse populations with a broader focus on minority groups who also menstruate, respecting their particularities and circumstances, are warranted. Identifying multi‐faceted factors that contribute to health disparities in the menopause can improve public health recommendations by informing evidence‐based interventions.

## Author Contributions

All authors contributed to the conception and design of the study. Material preparation and data collection were performed by Susy P. Saraiva, Carolina V.R. D'Aurea and Cristina Susy P. Saraiva Luz. The analysis and the first draft of the manuscript were written by Susy P. Saraiva, and all authors commented on previous versions of the manuscript. Claudia R.C. Moreno supervised and assisted throughout the study. Elaine C. Marqueze assisted with study design, data analysis, and discussions. Jose Cipolla‐Neto coordinated the project, secured funding, provided the study intervention, and gave substantial input regarding study design, sample size, and methodology. Fernanda G. Amaral assisted with methodology, manuscript revisions, and laboratory support for the analysis of biological materials. All authors read and approved the final manuscript.

## Ethics Statement

This study was conducted in accordance with the principles of the Declaration of Helsinki. All study participants received detailed information and provided written informed consent prior to participation. Approval was granted by the Research Ethics Committee of the hospital where data collection was conducted (permit no. 5.658.804, 22 September 2022) and by the Research Ethics Committee of the School of Public Health, University of São Paulo (FSP/USP) (permit no. 5.468.644, 14 June 2022).

## Consent

Informed consent was obtained from all individual participants included in the study. No individual person's identifiable data are included in this manuscript.

## Conflicts of Interest

The authors declare no conflicts of interest.

## Supporting information


**Table S1:** Generalized linear model (GLM) of percentage difference in Climacteric Symptoms (Kupperman Menopausal Index). Model adjusted for age, sleep duration on days off at baseline, anxiety, stress and depression; factors: shift, group, and menopausal status (*n* = 46). **Table S2**: Baseline sleep quality on days off and workdays, by group and work shift (*n* = 46). **Table S3**: Generalized linear model (GLM) of percentage difference in PSQI scores on days off, adjusted for age. **Table S4:** Generalized linear model (GLM) of percentage difference in LH, FSH, E2, and Progesterone levels (*n* = 46).

## Data Availability

The data that support the findings of this study are available from the corresponding author upon reasonable request.
