# Supplementary material for: Low‐Dose Melatonin, Climacteric Symptoms and Sleep in Female Shift Workers: A Randomized Controlled Trial
Source: J Pineal Res. 2026 Mar 17;78(2):e70140. doi: 10.1111/jpi.70140 (PMC12993913; doi:10.1111/jpi.70140)
Supplement: Supplementary file 1 — Table S1: Generalized linear model (GLM) of percentage difference in Climacteric Symptoms (Kupperman Menopausal Index). Model adjusted for age, sleep duration on days off at baseline, anxiety, stress and depression; factors: shift, group, and menopausal status (n = 46). Table S2: Baseline sleep quality on days off and workdays, by group and work shift (n = 46). Table S3: Generalized linear model (GLM) of percentage difference in PSQI scores on days off, adjusted for age. Table S4: Generalized linear model (GLM) of percentage difference in LH, FSH, E2, and Progesterone levels (n = 46). [file JPI-78-e70140-s001.docx]

**Supplementary material**

**Table S1** Generalized linear model (GLM) of percentage difference in Climacteric Symptoms (Kupperman Menopausal Index). Model adjusted for age, sleep duration on days off at baseline, anxiety, stress and depression; factors: shift, group, and menopausal status (n=46).

|  | **Mean (%)** | **SE** | ***p-value*** |
| --- | --- | --- | --- |
| **Group** |  |  |  |
| *Intervention* | -15.8 | 19.5 | **0.01*** |
| *Placebo* | 33.5 | 15.5 |  |
| **Shift** |  |  |  |
| *Morning* | 2.63 | 18.7 | 0.13 |
| *Afternoon* | -10.5 | 22.5 |  |
| *Night* | 34.4 | 18.3 |  |
| **Menopausal Status** |  |  |  |
| *Yes* | 11.7 | 20.4 | 0.78 |
| *No* | 5.93 | 15.5 |  |
| **Group x Menopausal Status** |  |  |  |
| *Intervention menopause* | -34.51 | 30.4 | **0.05*** |
| *Placebo menopause* | 58.04 | 21.2 |  |

Table S2 presents the results of a generalized linear model (GLM) analyzing the percentage difference in climacteric symptoms, as measured by the Kupperman Menopausal Index. The model was adjusted for potential confounders, including age, baseline sleep duration on days off, anxiety, stress, and depression. Fixed factors included work shift, groups (intervention vs. placebo), and menopausal status. *p<0.05. **p<0.01.

**Table S2**. Baseline sleep quality on days off and workdays, by group and work shift (n=46).

|  | Morning shift (n=16)  Mean ± SD | | *p* value | Afternoon shift (n=15)  Mean ± SD | | *p* value | Night shift (n=15)  Mean ± SD | | *p* value | *p*  *value*^a^ |
| --- | --- | --- | --- | --- | --- | --- | --- | --- | --- | --- |
|  | Intervention  group  (n=7) | Placebo group  (n=9) |  | Intervention  group  (n=8) | Placebo group  (n=7) |  | Intervention  group  (n=7) | Placebo group  (n=8) |  |  |
| Day-off sleep quality | 6.85 ±2.3 | 8.33 ±4 | n.s | 8.37±3.5 | 5.14±2.8 | n.s | 7.14±3 | 6.5±3.4 | n.s | 0.73 |
| Workday sleep quality | 6 ±2 | 7.55 ±3.1 | n.s | 8.62 ±3.4 | 5.86 ±2.4 | n.s | 9.83 ±3.2 | 8.25 ±4 | n.s | 0.12 |

Values are presented as mean ± (SD) for sleep quality scores on workdays and days off, in the intervention and placebo groups, stratified by work shift (morning, afternoon, night). Comparisons were performed using a Generalized Linear Model with group and shift as factors. p-value^a^ refers to the main effect of the shift factor alone. *p< 0.05, **p< 0.01, n.s. = not significant.

**Table S3**. Generalized linear model (GLM) of percentage difference in PSQI scores on days off, adjusted for age.

|  | **Mean (%)** | **SE** | ***p*-value** |
| --- | --- | --- | --- |
| **Group (G)** |  |  |  |
| *Intervention* | -35.33 | 6.33 | **< 0.001**** |
| *Placebo* | -4.09 | 5.25 |  |
| **Shift (S)** |  |  |  |
| *Morning* | -32.46 | 6.42 | **0.023*** |
| *Afternoon* | -19.31 | 8.46 |  |
| *Night* | -7.36 | 6.52 |  |
| **Menopausal Status** |  |  |  |
| *Yes* | -19.0 | 7.30 | 0.868 |
| *No* | -20.5 | 4.78 |  |

Table S4 displays the results of a generalized linear model (GLM) evaluating the percentage difference in Pittsburgh Sleep Quality Index (PSQI) scores on days off. The model was adjusted for age and included group (intervention vs. placebo), work shift (morning, afternoon, night), and menopausal status as fixed factors. Results are expressed as mean percentage differences with standard errors (SE). *p<0.05. **p<0.01.

**Table S4.** Generalized linear model (GLM) of percentage difference in LH, FSH, E2, and Progesterone levels (n=46).

|  |  | Morning shift  (n=16) | | | | Afternoon shift  (n=15) | | | | Night Shift  (n=15) | | | |  |  |  | |
| --- | --- | --- | --- | --- | --- | --- | --- | --- | --- | --- | --- | --- | --- | --- | --- | --- | --- |
| Reproductive hormones | | Intervention  group  (n=7) | | Placebo  group  (n=9) | | Intervention group  (n=8) | | Placebo  group  (n=7) | | Intervention group  (n=7) | | Placebo  group  (n=8) | | *p*-value | | | |
|  |  | Mean | SE | Mean | SE | Mean | SE | Mean | SE | Mean | SE | Mean | SE | G | | | S |
| LH  (mIU/mL) | | 27.93 | 77.5 | 111.67 | 70.6 | 50.38 | 107.4 | 138.79 | 74.9 | -33.98 | 78.6 | -8.99 | 73.4 | 0.306 | | | 0.303 |
| FSH (mIU/mL) | | -30.7 | 100.4 | 144.1 | 91.4 | 29.3 | 139 | 41.7 | 97 | 92.9 | 101.8 | -28.1 | 95.0 | 0.791 | | | 0.962 |
| E2  (pg/mL) | | 20.8 | 98.5 | 27.6 | 89.6 | 113.6 | 136.4 | 84.4 | 95.2 | -46.7 | 99.8 | 63.5 | 93.2 | 0.719 | | | 0.684 |
| Progesterone (ng/mL) | | 685.3 | 362 | 39.6 | 329 | 380.1 | 501 | 429.2 | 349 | -189.9 | 367 | 86 | 342 | 0.721 | | | 0.375 |

LH: luteinizing hormone, FSH: follicle-stimulating hormone, E2: estradiol. Model: GLM with 3 factors: shift, group and menopausal status. Adjusted for age. G: Group. S: Shift. *p<0.05. **p<0.01.
